# A306 A CASE OF SCLEROSING CHOLANGITIS AS A COMPLICATION OF CAR-T THERAPY

**DOI:** 10.1093/jcag/gwad061.306

**Published:** 2024-02-14

**Authors:** B Zhao, H Kim, A Huang, A Jain, D Chahal, E Yoshida

**Affiliations:** Medicine, The University of British Columbia Faculty of Medicine, Vancouver, BC, Canada; Medicine, The University of British Columbia Faculty of Medicine, Vancouver, BC, Canada; Medicine, The University of British Columbia Faculty of Medicine, Vancouver, BC, Canada; Medicine, The University of British Columbia Faculty of Medicine, Vancouver, BC, Canada; Medicine, The University of British Columbia Faculty of Medicine, Vancouver, BC, Canada; Medicine, The University of British Columbia Faculty of Medicine, Vancouver, BC, Canada

## Abstract

**Background:**

Chimeric antigen receptor T-cell (CAR-T) therapy is a novel immunotherapy for treating hematological malignancies. CAR-T therapy starts with the collection of T-cells from patients. Then, receptors on the T-cell membrane are re-engineered to become chimeric antigen receptors that specifically target antigens on the surface of malignant cells. These engineered cells are grown ex vivo and infused back into the patient, leading to a targeted immune response against cancer. While CAR-T therapy has shown promising results in treating malignancies refractory to conventional treatment, there have also been reports of complications from unwanted off-target effects with CAR-T therapy.

**Aims:**

We report a case of sclerosing cholangitis secondary to CAR-T therapy for the treatment of mantle cell lymphoma.

**Methods:**

Case report.

**Results:**

A 67-year-old male with refractory stage IVB mantle cell lymphoma underwent CAR-T therapy. His lymphoma was first diagnosed in 2011 and previous treatment included R-CHOP followed by autologous stem cell transplant. He had disease progression despite rituximab maintenance therapy and was switched to ibrutinib therapy with good response until 2023.

In 2023, he was noted to have progression of his lymphoma. He subsequently underwent CAR-T therapy. His treatment was complicated by grade 2 cytokine release syndrome requiring two doses of tocilizumab. Other complications included immune effector cell-associated neurotoxicity syndrome leading to status epilepticus. He was intubated and required monitoring in the ICU however he was not in shock, nor did he require vasopressors.

Post-CAR-T therapy he developed an increase of his liver enzymes in a cholestatic pattern. Magnetic resonance cholangiopancreatography (MRCP) showed irregular biliary dilation with beading suggestive of sclerosing cholangiopathy. He subsequently underwent multiple endoscopic retrograde cholangiopancreatography (ERCP) with stent insertions but there were no improvements in his liver enzymes and jaundice. A liver biopsy was performed which showed large bile duct obstruction with extensive cholestasis and some evidence of non-caseating granulomatous inflammation. Given the timing of new sclerosing cholangiopathy post-CAR-T therapy along with multiple other complications related to CAR-T therapy, it was thought this patient developed sclerosing cholangitis secondary to CAR-T therapy. Due to worsening clinical status along with progression of mantle cell lymphoma, he was transitioned to comfort care.

**Conclusions:**

CAR-T therapy is a novel immunotherapy for the treatment of refractory hematological malignancies. However, complications from the off-target effects of CAR-T therapy can occur. This case highlighted a rare side effect associated with CAR-T therapy. In the future, more research will be needed to further characterize the risks associated with CAR-T therapy.

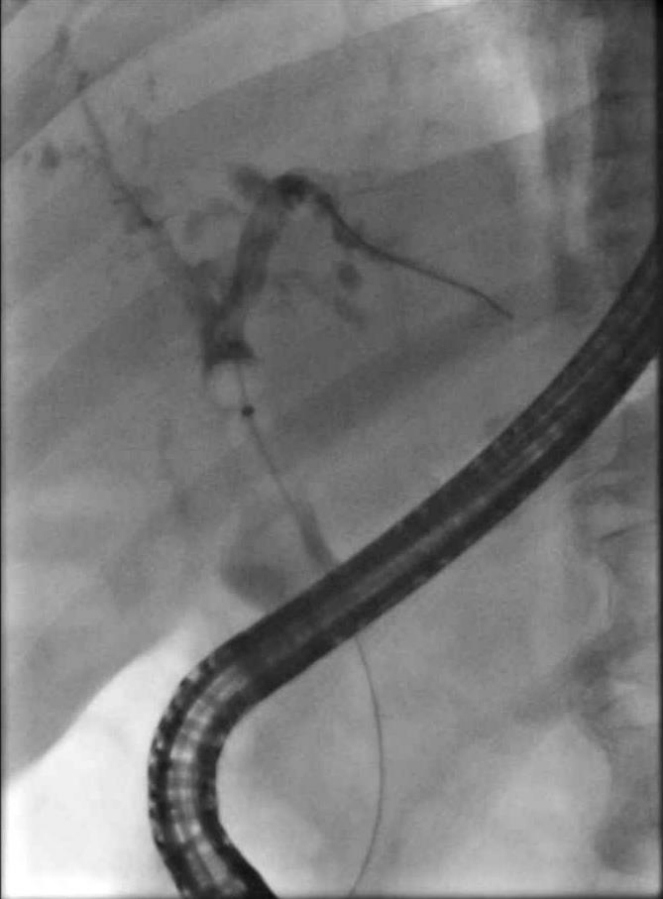

Cholangiogram showing no visible filling defects or obstructive lesion.

**Funding Agencies:**

None

